# Neurobehavioural Effects of the Methylimidazolium Ionic Liquid M8OI in Rats

**DOI:** 10.3390/jox16030113

**Published:** 2026-06-17

**Authors:** Tarek M. Abdelghany, Alaa A. Budastour, Ahmed S. Kamel, Sherehan M. Ibrahim, Alex Charlton, Simon Wilkinson, Catherine Arden, Noha F. Abdelkader, Matthew C. Wright

**Affiliations:** 1 Department of Pharmacology and Toxicology, Faculty of Pharmacy, Cairo University, Kasr El-Aini St., Cairo 11562, Egypt; 2 School of Medicine, Medical Sciences and Nutrition, University of Aberdeen, Foresthill, Aberdeen AB25 2ZD, UK; 3 School of Biomedical, Nutritional and Sport Sciences, Newcastle University, Newcastle upon Tyne NE2 4HH, UK; 4 Translational and Clinical Research Institute, Level 4 Leech, Newcastle University, Newcastle Upon Tyne NE2 4HH, UK; 5 Department of Pharmacology and Toxicology, Faculty of Pharmacy and Drug Technology, Egyptian Chinese University, Gesr El Suez St., Cairo 11786, Egypt; 6 Department of Pharmacology and Toxicology, Faculty of Pharmacy, Modern University for Technology & Information, Cairo 11571, Egypt; 7 Faculty of Science, Agriculture and Engineering, Newcastle University, Newcastle upon Tyne NE1 8QB, UK; 8 Biosciences Institute, Newcastle University, Newcastle upon Tyne NE2 4HH, UK

**Keywords:** M8OI, ionic liquids, mitochondrial dysfunction, Complex I inhibition, SH-SY5Y cells, neurobehaviour

## Abstract

M8OI is a cytotoxic methylimidazolium ionic liquid solvent through its binding to the ubiquinone binding site on complex I of the mitochondrial electron transport chain. Given the overlap in terms of toxic mechanism of action with the pesticide rotenone, the potential neurotoxic effects of M8OI were examined. In vitro, cytotoxicity and mitochondrial function were assessed in SH-SY5Y cells by measuring MTT reduction and oxygen consumption/extracellular acidification using a Seahorse analyser. SH-SY5Y cells were sensitised to M8OI toxicity by replacing medium glucose with galactose. Glucose protected the cells from M8OI toxicity, whereas galactose showed no clear dose–response protection. M8OI induced a dose-dependent reduction in oxygen consumption rate with a compensatory increase in extracellular acidification rate, consistent with inhibition of mitochondrial oxidative phosphorylation and a shift toward glycolysis. In vivo, rats were orally exposed via drinking water for 20 weeks and assessed using behavioural tests. In addition, the concentrations of M8OI and its metabolites were quantified by LC–MS in rat brain and other tissues. In rats, M8OI concentrations were ~30-fold higher in kidney than brain, and brain levels were at least 100-fold lower than the concentrations that affected SH-SY5Y cell viability in vitro. However, based on open field tests, M8OI exposure suppressed motor activity without any anxious behaviours. The cytotoxicity of M8OI in SH-SY5Y neuroblastoma cells was associated with metabolic mitochondrial dysfunction. However, the neurobehavioural changes observed in orally exposed rats occurred at significantly lower brain concentrations than would be predicted to lead to neural cell death. Nevertheless, direct comparisons between acute in vitro exposures and chronic in vivo outcomes should be interpreted cautiously.

## 1. Introduction

**Figure 1:**
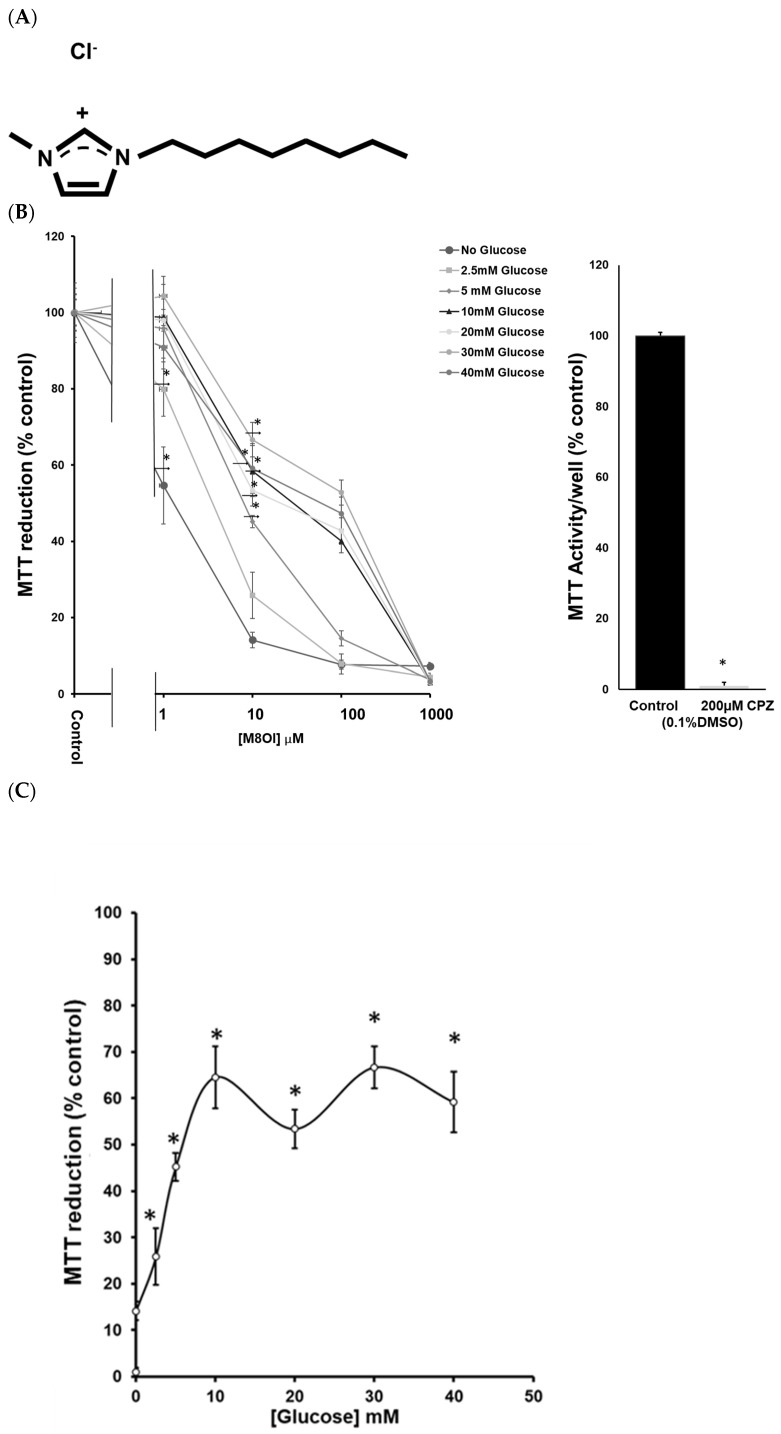

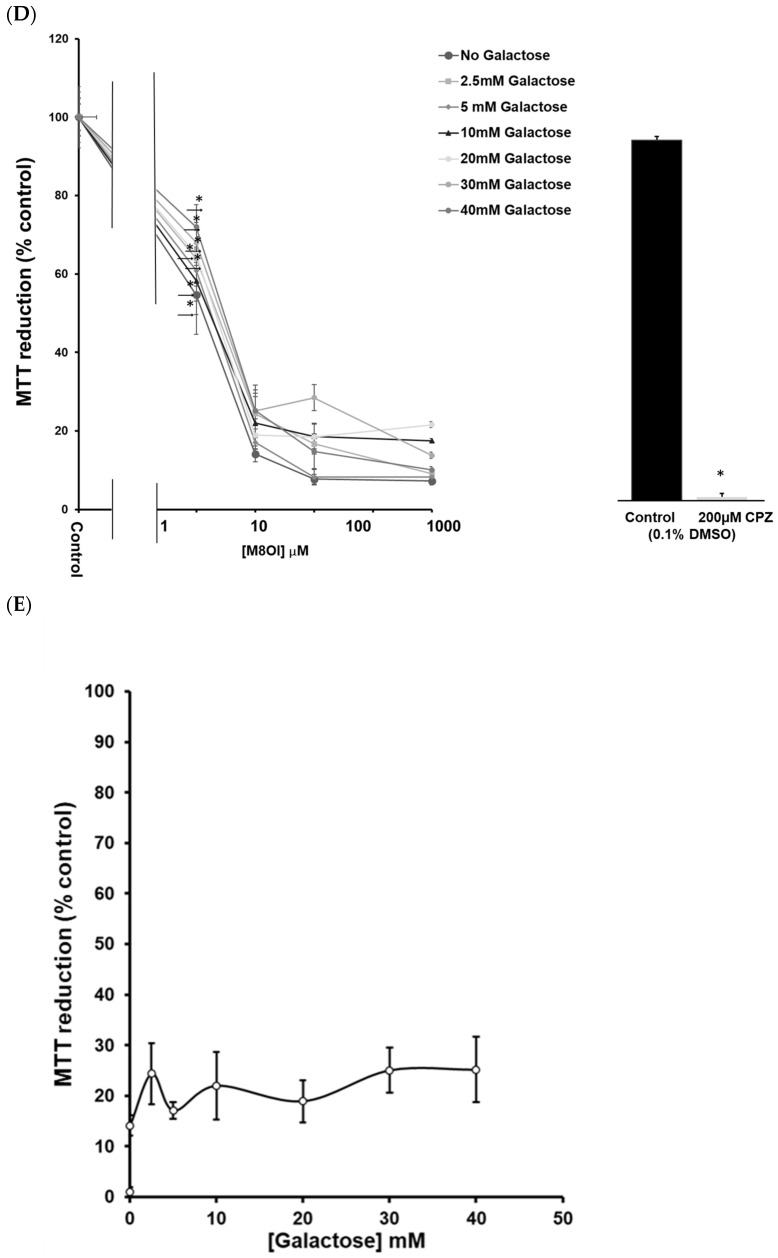
**M8OI toxicity in SH-SY5Y cells is abrogated by increasing medium glucose but not galactose concentrations**. (**A**), Chemical structure of M8OI. (**B**), SH-SY5Y cells were treated for 24 h with the indicated concentrations of M8OI in media containing varying glucose concentrations (0–40 mM), and MTT reduction was measured as an index of cell viability. CPZ, chlorpromazine. (**C**), Cells were exposed to 10 μM M8OI for 24 h in the presence of increasing glucose concentrations (0–40 mM). (**D**), Replacement of glucose with galactose (0–40 mM) increased cellular sensitivity to M8OI. (**E**), SH-SY5Y cells were exposed to 10 μM M8OI for 24 h in the presence of increasing galactose concentrations (0–40 mM). For each glucose/galactose condition, the matched −M8OI wells served as the glucose-only/galactose-only controls used for normalisation. Data are from *n* = 3 independent experiments, with 6 wells per condition per experiment. * Significantly different from control (two-tailed, *p* < 0.05), using one-way ANOVA followed by Dunnett’s multiple comparison test. Arrows indicate that significance applies from the indicated concentration onwards, including all points to the right.

1-octyl-3-methylimidazolium (M8OI) is a man-made methylimidazolium ionic liquid (for structure, see Figure 1A). Ionic liquids are a structurally diverse range of salts with low volatilities that are often liquids at ambient temperatures [1]. A major driver for their development is their potential use as “environmentally-friendly” solvents for use in various industrial and analytical applications [2,3], as well as pharmaceutical applications [4,5]. Recent studies have highlighted the potential of ionic liquids in oral drug delivery, transdermal delivery systems and in enhancing the solubility of poorly bioavailable drugs [4]. However, it has also been reported that certain ionic liquids, despite their delivery advantages, may cross biological barriers and accumulate in sensitive organs such as the brain, raising concerns about long-term safety in pharmaceutical use [5].

The M8OI cation has been shown to be present in the environment [6,7] and detectable in human serum [8,9], indicating exposure to the human population. M8OI is absorbed from the GI tract in mice after oral exposure [8]. It is oxidised on the alkyl chain to 1-(7-carboxyheptyl)-3-methyl-1H-imidazol-3-ium (COOH7IM) with little evidence for any additional pathways of metabolism or further metabolism (such as phase II pathways) [6,7]. It has been shown more recently that in humans, the monooxygenation of M8OI is primarily mediated by CYP3A4 and CYP3A5 [9]. In all systems examined thus far—B-13 rat cells [10]; human hepatocytes [9] and human HepaRG cells [11]—metabolism of M8OI is associated with a loss in toxicity, indicating that the parent M8OI molecule is the primary toxic species. In mice, M8OI has low systemic availability after oral exposure [8]. However, both parent M8OI and its metabolites are found at high levels in urine and bile; the former confirms systemic M8OI exposure to some extent [8].

Following acute i.p. administration in mice (two doses of up to 10 mg/kg bw given at 0 and 18 h, with cull at 24 h), renal injury was observed, ranging from mild focal to moderate multifocal degeneration, with a general increase in severity at higher doses [12]. These renal effects were accompanied by dose-dependent increases in kidney injury molecule-1 (Kim1) expression in kidney tissue, marked elevations in urinary Kim1 protein content, and increased serum creatinine [12]. Hepatic effects were more limited and included a significant dose-dependent depletion of hepatic glycogen and a mild but significant increase in portal tract inflammatory recruitment and fibroblastic proliferation, with focal fibrotic change [12]. Notably, no overt pathological changes were observed in the heart or brain of mice [12]. Likely, reflective of its low oral bioavailability, it took 18 weeks of oral exposure via drinking water (440 mg/L drinking water for 18 weeks, estimated to be equivalent to 66 mg M8OI/kg bw per day) to observe mild degenerative changes in the kidney with only glycogen depletion as the observed effect in the liver in mice [8].

However, cardiac toxicity (based on histopathology and clinical chemistry endpoints) was seen after oral M8OI exposure in rats (880 mg/L drinking water for 20 weeks; estimated to be equivalent to 44 mg/kg bw per day) [13]. Expression-based analyses further suggested that mice may exhibit reduced sensitivity to methylimidazolium ionic liquid(s) (MILs)-driven cardiotoxicity, potentially reflecting greater breast cancer resistance protein (BCRP)-mediated efflux and excretion of M8OI from cardiac cells [13].

In terms of toxicity at the cellular level, in most cell types in vitro, M8OI induces an apoptotic mode of cell death in mammalian cells [14,15,16]. In our hands, the earliest effect (within minutes) observed in cells exposed to M8OI is an inhibition of oxygen consumption [6,10,17]. This effect appears to be primarily dependent on M8OI binding to the ubiquinone binding site on complex I of the electron transport chain [18]. In this respect, this interaction is identical to the insecticide/piscicide/pesticide—rotenone—and, similarly to rotenone-treated cells, is accompanied by a production of reactive oxygen species (ROS) and oxidative stress [18]. Given that inhibition of complex I is a recognised initiating event in rotenone-associated neurotoxicity and Parkinsonian models, this provides a rationale for considering whether M8OI might be associated with neurobehavioural effects in vivo.

Parkinson’s disease (PD) is a progressive neurological disorder in which degeneration of the substantia nigra is associated with reduced dopamine levels in the brain [19]. The reduction in dopamine levels accounts for defective control of muscle movements seen in PD, amongst other effects [19]. The causes of PD are unknown but thought to be dependent on genetic susceptibility in combination with environmental effects [20]. Xenobiotics—in particular pesticides—are included as one of the environmental factors thought to contribute to PD development [21,22,23].

Recent work has demonstrated that exposure to low-dose [C8mim][PF6] (1-octyl-3-methylimidazolium hexafluorophosphate) induces neurotoxicity and behavioural abnormalities in young rats [24]. Consistent with this, long-term exposure to another [C8mim] salt ([C8mim]Br) has also been reported to increase the risk of anxiety-like behaviour and memory deterioration, with associated disturbance of neurotransmitter systems [25]. However, the complexity of examining the potential toxicity of ionic liquids is that they comprise at least 2 moieties, which could each alone have adverse effects, with a possibility also of each potentiating the toxicity of the other. To consider only the potential toxicity of the [C8mim] cation, we restrict investigations to the Cl^−^ salt (i.e., M8OI).

Since rotenone has been shown to induce PD-like effects in rats [26,27] and C8mim.PF6 has been shown to be neurotoxic in developing rats [24], it was hypothesised that M8OI may be a hazard for neurotoxic effects. To test this hypothesis, the effects of M8OI were initially examined in a human neuroblastoma cell line. These studies were followed by oral administration of M8OI to rats, for assessment of their neurological function. This study provides neurobehavioural evidence following chronic oral exposure to M8OI, together with in vitro mitochondrial bioenergetic assessment and tissue quantification of M8OI and metabolites and discusses the apparent disconnect between low measured brain concentrations and behavioural outcomes in the context of chronic exposure.

## 2. Materials and Methods

### 2.1. Materials

M8OI (purity > 97%) was sourced from Merck (Poole, UK). Hydroxylated (HO8IM) and carboxylated (COOH7IM) metabolites of M8OI (purity > 98%) were previously custom synthesised according to published methods [6,7]. All other chemicals, unless otherwise stated, were purchased from Merck (Poole, UK).

### 2.2. Cell Culture

Human undifferentiated SH-SY5Y neuroblastoma cells (ATCC; CRL-2266™) were purchased from the American Type Culture Collection (ATCC) via LGC, Guildford, UK. SH-SY5Y cells were used as a neuronal model for mechanistic screening, including in MTT reduction assays under defined substrate conditions (glucose vs. galactose) and in OCR/ECAR bioenergetic assessments in Seahorse assay medium, as a practical and 3Rs-aligned approach prior to further primary neuronal or in vivo work. Human undifferentiated SH-SY5Y neuroblastoma cells were routinely cultured in RPMI (high glucose, 25 mM) supplemented with 10% (*v*/*v*) fetal calf serum (FCS), 80 units/mL penicillin, 80 μg/mL streptomycin and 0.584 g/L l-glutamine. Cells were cultured at 37 °C in a humidified incubator with 5% CO_2_ in air. Where required, test compounds were added from vehicle-solvated stock solutions prepared at 1000× concentration.

### 2.3. MTT Reduction Assays

Thiazolyl blue tetrazolium bromide (methylthiazolyldiphenyl-tetrazolium bromide, MTT) reduction was used as a proxy measure of cell viability/metabolic competence, essentially as described previously [28]. Briefly, MTT (0.5 mg/mL) was added to cells and incubated under standard culture conditions for 1 h. The medium was then removed and replaced with an equal volume of isopropanol to solubilise the formazan product. After mixing, absorbance was measured at 570 nm, with background correction at 690 nm. Given the mitochondrial mode of action of M8OI, MTT reduction was interpreted as an integrated readout of metabolic competence and viability.

For MTT reduction assays, SH-SY5Y cells were exposed to M8OI at concentrations ranging from 1 µM to 1000 µM for 24 h. Each experiment was repeated *n* = 3 independent times, with 6 wells per condition. All treatment groups were normalised to the MTT reduction of non-exposed (vehicle-treated; −M8OI) control cells, which was set to 100%.

### 2.4. Seahorse Assays

OCR and extracellular acidification rate (ECAR) were measured in intact SH-SY5Y cells using a Seahorse XFe96 analyser (Agilent, Stockport, UK), in accordance with the manufacturer’s instructions. Sensor cartridges were hydrated in deionised water for 24 h prior to the experiment at 37 °C at ambient air CO_2_ levels and the water was replaced with the Seahorse XF Calibrant 40 min prior to the experiment. One day before the experiment, the SH-SY5Y cells were cultivated in the 96-well Seahorse XF cell culture microplates (Agilent, Stockport, UK) at a density of 20,000 cells per well in their normal culture medium (RPMI-1640 medium as described above). The cells were washed twice one hour before the experiment with the seahorse assay medium containing (Seahorse XF Base Medium (Agilent, Stockport, UK), freshly supplemented with 5.5 mM glucose, 2 mM glutamine, 1 mM pyruvate and the pH was adjusted to 7.4) and 180 μL assay medium was added to each well and cells were then incubated for one hour in the same incubator with the hydrated cartridge (37 °C at ambient air CO_2_ levels). After recording basal respiration using a 2-3-2 min (mix-wait-measure) cycle, compounds of interest were added and cells were monitored for 1 h using a 3-14-3 min (mix–wait–measure) cycle. This was followed by three 2-3-2 min measurement cycles after sequential injections of oligomycin (1 μM), carbonyl cyanide-p-trifluoromethoxy phenylhydrazone (FCCP, 1 μM), and rotenone/antimycin A (0.5 μM/0.5 μM) (all compounds from Agilent, Stockport, UK), consistent with established approaches for OCR/ECAR-based bioenergetic profiling and interpretation [29,30]. OCR and ECAR values were normalised to protein content per well at the end of the experiment, determined using the Bradford reagent (Merck, Poole, UK). Data were analysed using Seahorse Wave software (v2.6.0).

For Seahorse assays, SH-SY5Y cells were exposed to M8OI at 1–1000 µM during real-time OCR/ECAR measurements. Each experiment was repeated *n* = 3 independent times, with 6 wells per condition.

### 2.5. Disposition of M8OI in Orally Exposed (Drinking Water) Male Rats

Male Wistar rats (180–220 g, 16 weeks old), originally bred at the animal facility of the Faculty of Pharmacy (Cairo University, Cairo, Egypt), were housed and maintained at the same facility throughout the study period. Animals were kept in an air-conditioned environment (25 ± 2 °C; 60 ± 10% humidity) under a 12 h light/dark cycle, with ad libitum access to food and water. To reduce animal use, tissues from a previous rat study [13] in which rats were exposed to M8OI via drinking water were used. In this study, Wistar rats (180–220 g body weight) were randomly allocated into cages (3–4 rats per cage) and two cages were randomly assigned to either control (2 cages, 7 rats; normal tap drinking water) or M8OI (2 cages, 7 rats; tap drinking water containing 880 mg/L M8OI). Rats were treated for 20 weeks and the M8OI dose was estimated to be equivalent to 44 mg/kg bw per day [13,31]. This dose was selected at the time because mice previously readily tolerated M8OI in drinking water at 440 mg/L [8] and a preliminary study in rats with M8OI (0, 220, 440, 880 mg/L drinking water for 4 weeks) was readily tolerated at the highest dose [13]. As a qualitative study, only male rats and a single dose were used at this time, to reduce the number of animals used and to avoid potential complications of variations in sex hormone levels seen in females. Since the aim of this study was to identify potential neurotoxicological hazards, the dose was selected based on the preliminary 4-week drinking water study, which showed no mortality/overt adverse effects. M8OI has been detected in human serum; however, population exposure distributions and reference exposure levels are not established for formal dose selection. Tissue quantification and behavioural endpoints were derived from separate cohorts; therefore, comparisons are contextual rather than a direct PK/PD linkage. Due to limited remaining tissue availability from this prior study, liquid chromatography–tandem mass spectrometry (LC–MS/MS) tissue quantification was performed on a subset of animals (*n* = 3 per group).

### 2.6. LC/MS MS Determination of M8OI and Its Metabolites in Rat Brain, Kidney and Serum

Brain and kidney tissues and serum from a previous study [13] were snap-frozen in dry ice at termination and stored at −80 °C until screening for the presence of M8OI. To maintain analyte stability and minimise handling-related variability, samples were thawed on ice immediately prior to preparation and processed promptly (including ice-cooled perchlorate protein precipitation and centrifugation at 4 °C), and extracts/supernatants were kept at 4 °C and analysed without unnecessary delay. Only limited remaining tissue was available for LC–MS/MS screening and quantification; therefore, tissue analysis was performed on *n* = 3 treated animals and *n* = 3 control animals. Whole brain homogenate was used for quantification (i.e., the analysis was not region-specific). Frozen brain and kidney tissues were defrosted, and approximately 100 mg from each was weighed and homogenised by sonication in ice-cooled STIM buffer (STIM buffer was prepared to contain 0.10 M NaCl, 5.4 mM KCl, 0.34 mM Na_2_HPO_4_·12H_2_O, 0.44 mM KH_2_PO_4_, 20 mM glucose, 1 mM CaCl_2_, 40 mM NaHCO_3_, and 4 mM glutamine, and was further supplemented with 100 μM each of L-alanine, L-asparagine, L-aspartic acid, L-glutamic acid, glycine, L-proline, and L-serine). The buffer was adjusted to pH 7.4 by gassing with 5% CO_2_ in air to prepare a 10% *w*/*v* homogenate. This was done by adding 100 mg of tissue to ~0.8 mL of STIM buffer. After sonication, 100 µL of 600 mM Na perchlorate was added to each 900 µL tissue homogenate. Samples were then centrifuged at 13,000 rpm for 10 min at 4 °C. The supernatant was collected and used for analysis. Serum samples were processed by adding 10 µL of 600 mM Na perchlorate to 90 µL of serum. Supernatants and serum samples were diluted with HPLC-grade water before analysis. M8OI and its hydroxylated and carboxylated metabolites were quantified using standard multiple reaction monitoring (MRM) on a Waters Xevo TQ-S triple quadrupole mass spectrometer fitted with a Waters BEH-C18 column (100 × 2.1 mm, 1.7 μm particle size), following the approach described previously [32]. Chromatographic separation was achieved by gradient elution using solvents; A (0.1% formic acid in water) and B (0.1% formic acid in acetonitrile) at a flow rate of 250 μL/min. The gradient was set to 5% B at t = 0, increased to 30% B at 5 min and to 95% B at 8 min, maintained at 95% B until 9 min, then returned to 5% B at 9.5 min and held at 5% B until 12 min. The column temperature was maintained at 40 °C, and samples were injected at 1 μL per run.

### 2.7. Neurobehavioural Effects of M8OI in Orally Exposed (Drinking Water) Male and Female Rats

Twenty male and twenty female Albino Wistar rats were randomly allocated into eight cages (five rats per cage), and cages were randomly assigned to one of two treatment groups (Control or M8OI), with two cages per treatment per sex. The experimental unit was the individual animal. Under the same environmental conditions (temperature, humidity, and light–dark cycle), the positions of the cages were randomised within the animal facility. The randomisation was achieved using a random number generator to overcome any allocation bias. The sample size was determined based on previous studies and confirmed using G*Power software (version 3.1, Düsseldorf, Germany). The power analysis utilised an effect size of 0.6, an alpha level of 0.05, and a statistical power of 0.8. The locomotor activity was the primary outcome measure used for sample size determination, observed in total distance travelled in the open field test. Rats received either normal tap water (control group) or tap water containing 880 mg/L M8OI, estimated to be equivalent to 44 mg/kg bw per day in both males and females [13,31]. Body weight was measured weekly throughout the 20-week exposure period, and no meaningful differences were observed between control and M8OI-treated groups. Water intake was not recorded; therefore, the daily M8OI intake is presented as an estimated dose based on drinking water concentration rather than a measured individual intake. By the end of the 20th week, rats were trained and tested for potential neurobehavioural changes [open field test (OFT), rotarod, and elevated plus maze (EPM) tests]. To minimise potential confounders related to test order and stress, behavioural testing was conducted in the following sequence: OFT, rotarod, then EPM. For all groups, treatments and behavioural assessments were conducted in a consistent order and at standardised times. These procedures were conducted in a blinded manner for neurobehavioural assessment and data analysis, with data analysis performed by third-party investigators unaware of group assignments. No animals or data points were excluded from analysis.

### 2.8. Open Field Test (OFT)

The OFT was conducted in a square wooden arena (80 × 80 × 40 cm) with red walls and a smooth, polished white floor marked by black lines into 16 equal squares (20 × 20 cm). Each rat was placed in the centre of the arena and allowed to explore freely for 3 min. The floor and walls were cleaned between animals to minimise any potential bias from odours left by previous rats. A camera mounted above the arena recorded movement and behaviour, which were analysed using ANY-Maze video tracking software (Stoelting Co, Wood Dale, IL, USA). Total distance travelled (OFT-D), mean speed (OFT-MS), time immobile (OFT-TI), centre time (OFT-CT), centre distance travelled (OFT-TD), and thigmotaxis time (OFT-TGX) were recorded [20].

### 2.9. Rotarod Test

The rotarod test was performed using an automated 5-lane rotarod apparatus (Model 47750, Ugo Basile, Gemonio, Italy) accelerating from 4 to 40 rpm. Each rat completed three trials, with a 5 min cut-off time and rest period between trials. The mean rotarod fall-off latency (RR-FOL) was calculated for each animal. Rats were trained for two consecutive days prior to testing at a constant speed of 4 rpm, and only animals able to remain on the rod for 5 min were included in the test.

### 2.10. Elevated Plus Maze (EPM) Test

EPM test was performed in a room illuminated to 400 lux in an apparatus comprising four arms (10 cm wide by 50 cm long); two enclosed by 40 cm tall walls (the “closed arms”) and two open (the “open arms”). The height of the apparatus from the floor was 60 cm. Each rat was placed in the centre of the maze, facing one of the closed arms, and allowed to explore freely for 5 min. After each trial, the apparatus was cleaned with an ethanol solution and allowed to dry before the next animal was tested. The number of entries (all 4 paws enter the arm) into open and closed arms was recorded. Frequency of unprotected stretch-attend postures and head dips (animal sticking the head outside the maze border and toward the floor) was also recorded.

### 2.11. Data Handling and Statistical Analysis

Data are presented as means or (mean and range) ± standard deviation (SD). Normality was assessed prior to parametric testing using the Shapiro–Wilk test in GraphPad Prism. For behavioural tests, comparisons between sex-matched control and M8OI-treated groups were carried out using an unpaired two-tailed *t*-test. For in vitro experiments involving comparisons between more than two groups, statistical analysis was performed using one-way analysis of variance (ANOVA) followed by Dunnett’s multiple comparison test (each concentration vs. the matched control condition). For all statistical tests, a probability level of significance of *p* < 0.05 was accepted as statistically significant. Statistical analysis was performed using GraphPad Prism^®^ software package, version 8 (GraphPad Software Inc., San Diego, CA, USA).

## 3. Results

### 3.1. Glucose Attenuates M8OI Toxicity in SH-SY5Y Cells, Whereas Galactose Sensitises Cells

Previous work in the human liver-like HepaRG cell has shown that M8OI is toxic and that glucose is protective to the cells [11]. Substituting glucose with galactose increased the sensitivity of HepaRG cells to M8OI [11].

SH-SY5Y cells are routinely propagated in high-glucose medium (25 mM). The cytotoxicity of M8OI was therefore evaluated across a range of glucose concentrations representing low (0 and 2.5 mM), physiological (5 mM), and elevated conditions. As shown in Figure 1B, 24 h exposure to M8OI caused a dose-dependent decrease in MTT reduction in SH-SY5Y cells, with toxicity attenuated at higher glucose concentrations. In the absence of M8OI, glucose-free conditions reduced baseline MTT reduction; therefore, responses were normalised to the –M8OI control within each condition. For clarity, the effects of glucose or galactose alone were captured by the matched −M8OI controls at each substrate concentration, which served as the 100% reference within each condition for normalisation. Baseline (−M8OI) MTT reduction was lowest under glucose-free conditions (~0.83) and was higher across glucose-containing conditions (~1.33–1.64 at 5–10 mM glucose and ~1.41–1.45 at 20–40 mM glucose). Under galactose-only conditions, baseline (−M8OI) MTT reduction showed a lower range overall and decreased with increasing galactose at several concentrations (e.g., ~0.92 at 2.5 mM, ~0.67 at 5 mM, ~0.50 at 10 mM and ~0.38 at 20 mM galactose), before partially recovering at 30–40 mM (~0.65–0.57). Consistent with this, when cells were challenged with a moderately toxic dose of M8OI (10 µM), increasing glucose concentrations conferred dose-dependent protection (Figure 1C), with an EC_50_ of 83.3 µM at 20 mM glucose. In contrast, substituting glucose with galactose (0–40 mM) increased cellular sensitivity to M8OI (Figure 1D), with an EC_50_ of 6.1 µM at 20 mM galactose. Moreover, galactose did not provide dose-dependent protection against 10 µM M8OI (Figure 1E). Notably, the EC_50_ at 20 mM galactose was ~13-fold lower than at the same glucose concentration (6.1 vs. 83.3 µM), indicating markedly enhanced susceptibility under galactose conditions. Chlorpromazine (CPZ) was included as a comparator positive control compound and was used at 200 µM, a concentration within the cytotoxic range reported for CPZ in vitro (e.g., reduced viability in SH-SY5Y cells at 0.1 mM) [33].

### 3.2. M8OI Suppresses Mitochondrial Respiration with a Compensatory Increase in Glycolysis in SH-SY5Y Cells

**Figure 2:**
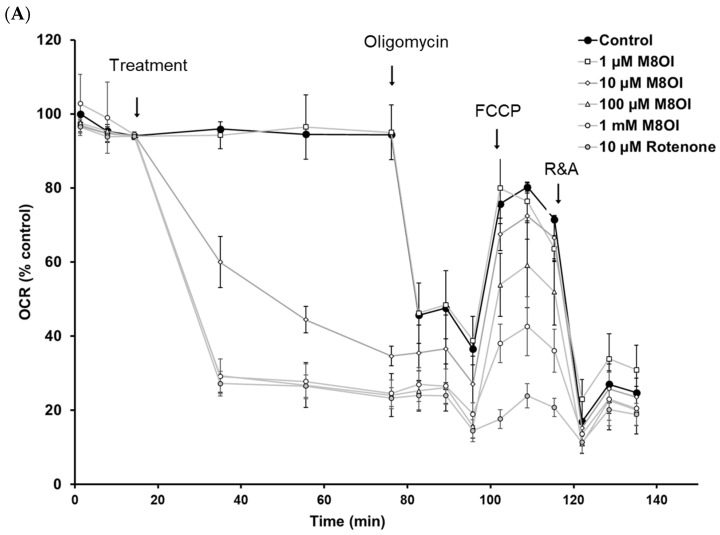

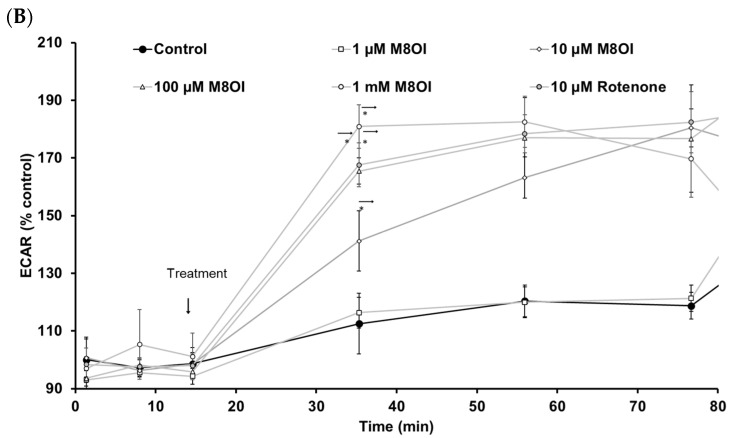
The effect of M8OI on mitochondrial oxidative phosphorylation and glycolysis in SH-SY5Y cells. SH-SY5Y cells were maintained in Seahorse XF assay medium supplemented with 5.5 mM glucose, 2 mM glutamine and 1 mM pyruvate, and exposed to the indicated concentrations of M8OI during real-time assessment of oxygen consumption rate (OCR; (**A**)) and extracellular acidification rate (ECAR; (**B**)). Data are from *n* = 3 independent experiments, with 6 wells per condition per experiment. * Significantly different (two-tailed, *p* < 0.05) from control using one-way ANOVA followed by Dunnett’s multiple comparison test (each concentration vs. control). Arrows indicate that significance applies from the indicated concentration onwards, including all points to the right.

M8OI inhibited OCR (Figure 2A) and increased ECAR (Figure 2B) in a dose-dependent manner in SH-SY5Y cells, consistent with a compensatory shift toward glycolytic metabolism. These effects were observed at concentrations ≥ 1 µM M8OI, with marked decreases in mitochondrial function at the 10 µM concentration, corresponding to concentrations that also cause significant reductions in cell viability (based on MTT reduction capacities).

These data suggest that M8OI is toxic to SH-SY5Y cells through a similar mechanism to that identified in other cell types from a variety of species (rat B-13 and B-13/H cells [6,10,18]; human HepaRG cells [11]), namely inhibition of mitochondrial oxidative phosphorylation, with protection from toxicity by glucose through compensatory increases in glycolysis [18,32,34]. Of note, the effects on OCR and ECAR in SH-SY5Y likely occur above a threshold of approx. 1 µM M8OI, with toxicity observed above a threshold between 1 and 10 µM M8OI. The concentrations producing OCR suppression in SH-SY5Y cells were within the low micromolar range reported for other cell types [17,18].

### 3.3. Tissue Levels of M8OI and Metabolites Following Oral Exposure in Rats

Tissue quantification and behavioural outcomes were derived from separate cohorts; therefore, the tissue concentrations provide contextual exposure information rather than a direct concentration–behaviour linkage within the same animals.

To consider whether the brain might be directly affected by oral exposure to M8OI, male rats exposed to M8OI in their drinking water (880 mg/L for 20 weeks, estimated to be equivalent to 44 mg/kg bw per day [31]) were examined for brain levels of M8OI or its metabolites.

Table 1 and Supplementary Figure S1 demonstrate that M8OI and its metabolites were detectable in rat brains. Brain levels of M8OI were around 3 times lower than circulating serum levels and markedly lower (approximately 30-fold) than kidney levels. Representative LC–MS chromatograms are provided in the Supplementary Material (Figure S1), whereas quantitative tissue concentrations are summarised in Table 1. Individual animal-level concentrations underlying Table 1 are provided in the Supplementary Material (Table S1).

### 3.4. M8OI Suppresses Locomotor/Exploratory Activity Without Marked Anxiogenic Behaviour

Body weight was monitored weekly over the exposure period, and no meaningful differences were observed between control and M8OI-treated groups.

These data indicate that total brain concentrations of M8OI in male rats were at least 100-fold lower than the medium concentrations of M8OI required to reduce SH-SY5Y mitochondrial function and cell viability (based on OCR and MTT reduction, respectively).

**Figure 3:**
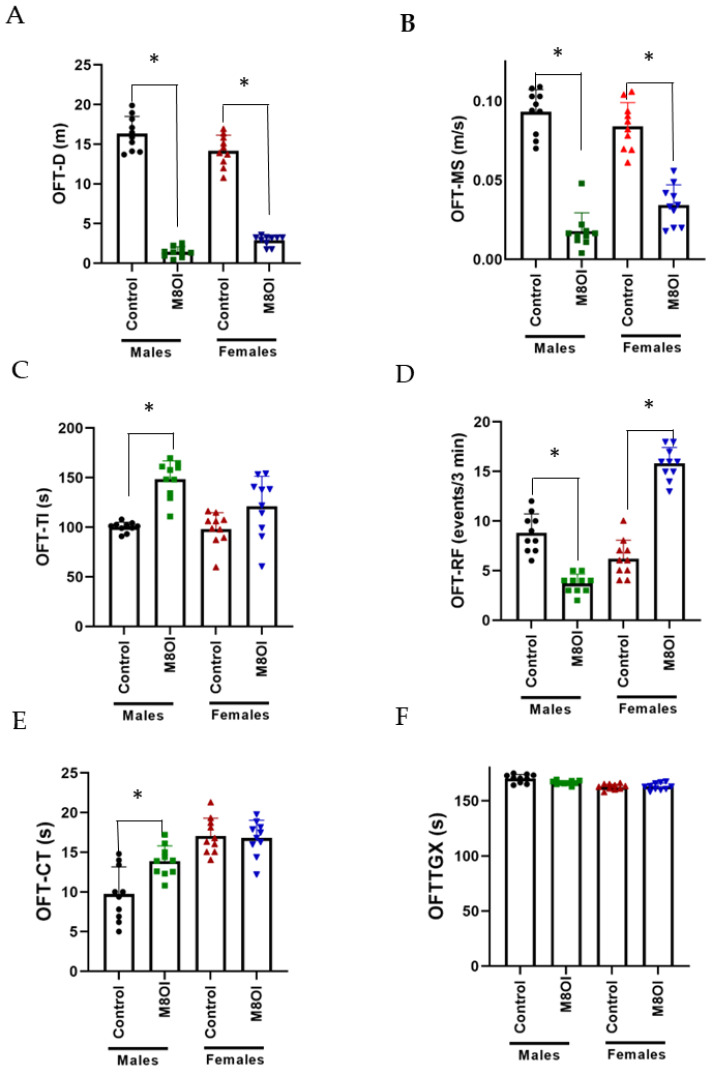
**Open field test (OFT) endpoints in rats orally exposed to M8OI. Albino Wistar rats received M8OI in drinking water (880 mg/L; estimated to be equivalent to 44 mg/kg bw/day) for 20 weeks and were assessed in the OFT for 3 min, with behaviour analysed using ANY-maze video tracking**. Panels show (**A**), total distance travelled (OFT-D, m), (**B**), mean speed (OFT-MS, m/s), (**C**), time immobile (OFT-TI, s), (**D**), rearing frequency (OFT-RF, events/3 min), (**E**), time spent in the centre (OFT-CT, s), and (**F**), thigmotaxis time (OFT-TGX, s). Data are mean ± SD (*n* = 10 per sex per treatment). * *p* < 0.05 versus sex-matched control (unpaired *t*-test). Abbreviations: OFT, open field test; OFT-D, open field test distance travelled; OFT-MS, open field test mean speed; OFT-TI, open field test time immobile; OFT-RF, open field test rearing frequency; OFT-CT, open field test centre time; OFT-TGX, open field test thigmotaxis time.

The open field test (OFT) is a widely used behavioural test to assess exploratory behaviour and general activity of rodents [35]. Figure 3 demonstrates that rats orally exposed to M8OI via their drinking water significantly reduced their exploratory and general activity.

In both males and females, there was a statistically significant reduction in distance travelled (OFT-D) and mean speed (OFT-MS) parameters in animals treated with M8OI. This corresponded to a 90.6% reduction in OFT distance travelled in males and an 80.0% reduction in females (Figure 3A), and an approximate 83.7% reduction in mean speed in males and a 62.6% reduction in females (Figure 3B), compared with sex-matched controls. A statistically significant increase in the time spent immobile (OFT-TI) and time spent in the centre (OFT-CT) was also seen in M8OI-treated males. This corresponded to a 50.1% increase in time immobile (Figure 3C) and a 48.9% increase in centre time (Figure 3E) in males compared with controls. Females also showed an increase in OFT-TI, but this was not statistically significantly different from control females. With respect to OFT-CT, M8OI did not affect females (consistent with only a negligible change in females; ~0.4% increase).

A clear sex difference was seen with regard to head rearing frequency (OFT-RF), where M8OI treatment resulted in a statistically significant reduction in males and a statistically significant increase in females. This corresponded to a 59.3% reduction in males and a 158.3% increase in females compared with sex-matched controls (Figure 3D). M8OI had no clear effect on thigmotaxis (OFT-TGX), movement in response to physical contact with surfaces (consistent with only small changes; ~2.5% reduction in males and ~0.2% increase in females).

Based on the OFT, M8OI treatment therefore suppressed motor activity without markedly affecting anxious behaviours. This conclusion is supported by both the rotarod (assess motor coordination and balance) and the elevated plus maze (EPM, assess anxiety to open spaces).

**Figure 4:**
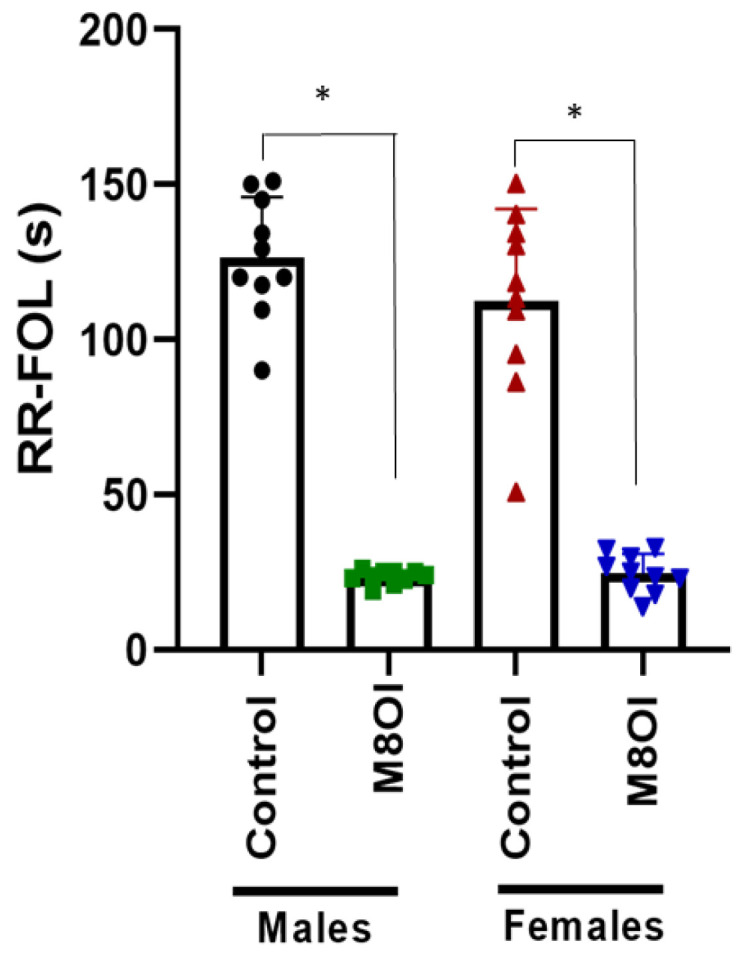
**Rotarod performance in rats orally exposed to M8OI. Albino Wistar rats received M8OI in drinking water (880 mg/L; estimated to be equivalent to 44 mg/kg bw/day) for 20 weeks and were assessed on the rotarod for motor coordination and balance**. Shown is rotarod fall-off latency (RR-FOL, s) for control and M8OI-exposed rats. Data are mean ± SD (*n* = 10 per sex per treatment). * *p* < 0.05 versus sex-matched control (unpaired *t*-test). Abbreviations: RR-FOL, rotarod fall-off latency.

Figure 4 demonstrates that M8OI treatment statistically significantly reduced motor coordination and balance in both males and females to a similar extent. This corresponded to an 81.7% reduction in RR-FOL in males and an 80.0% reduction in females relative to sex-matched controls (Figure 4).

**Figure 5:**
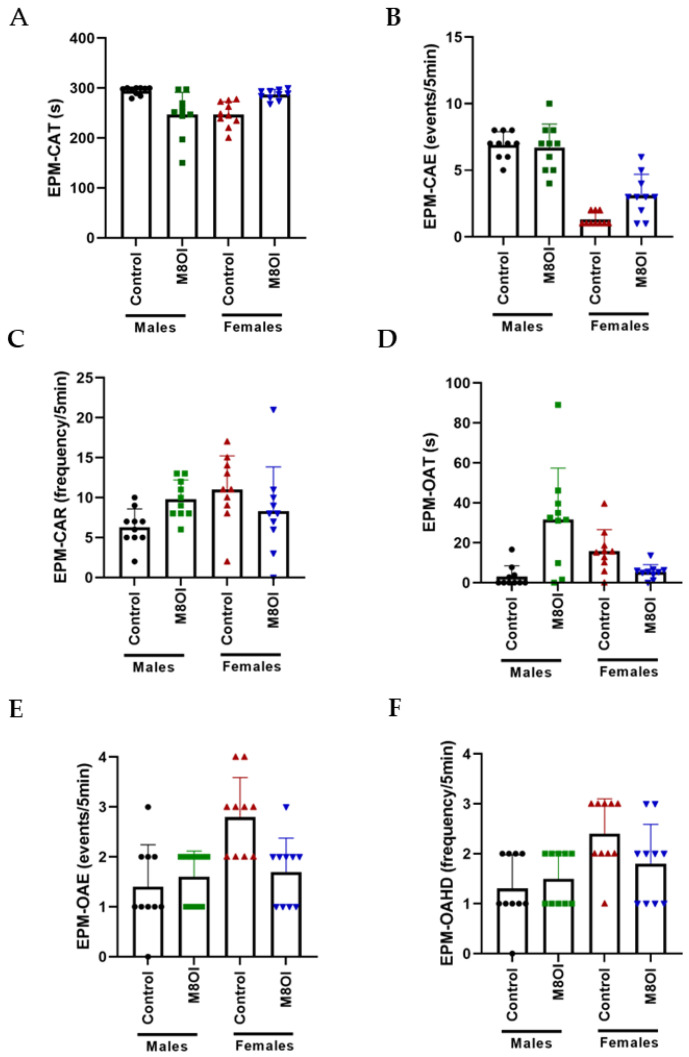

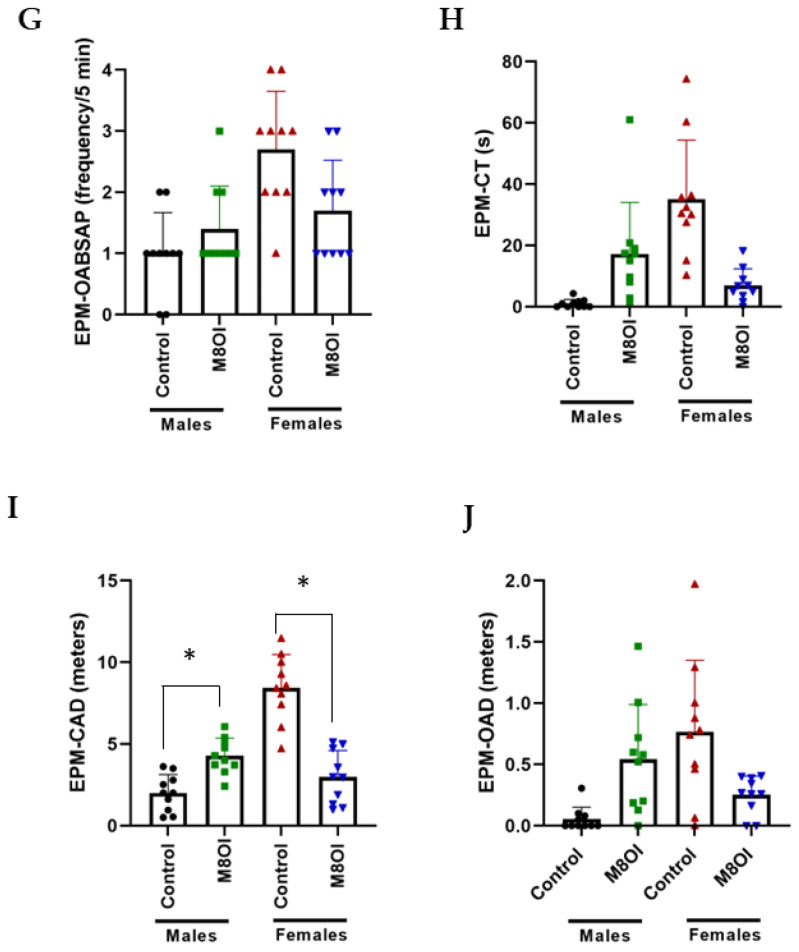
**Elevated plus maze (EPM) endpoints in rats orally exposed to M8OI. Albino Wistar rats received M8OI in drinking water (880 mg/L; estimated to be equivalent to 44 mg/kg bw/day) for 20 weeks and were assessed in the EPM for 5 min, with behaviour analysed using ANY-maze video tracking**. Panels show (**A**), closed arm time (EPM-CAT, s), (**B**), closed arm entries (EPM-CAE, events/5 min), (**C**), closed arm rearing (EPM-CAR, frequency/5 min), (**D**), open arm time (EPM-OAT, s), (**E**), open arm entries (EPM-OAE, events/5 min), (**F**), open arm head dipping (EPM-OAHD, frequency/5 min), (**G**), open arm body stretching/SAP (EPM-OABSAP, frequency/5 min), (**H**), centre time (EPM-CT, s), (**I**), closed arm distance travelled (EPM-CAD, m), and (**J**), open arm distance travelled (EPM-OAD, m). Data are mean ± SD (*n* = 10 per sex per treatment). * *p* < 0.05 versus sex-matched control (unpaired *t*-test). Statistical comparisons were performed within sex (sex-matched control vs. M8OI); male–female comparisons within control groups or within M8OI-exposed groups were not assessed statistically. Abbreviations: EPM, elevated plus maze; CAT, closed arm time; CAE, closed arm entries; CAR, closed arm rearing; OAT, open arm time; OAE, open arm entries; OAHD, open arm head dipping; OABSAP, open arm body stretching/SAP; CT, centre time; CAD, closed arm distance travelled; OAD, open arm distance travelled.

Figure 5 demonstrates that only one out of the 10 elevated plus maze (EPM) endpoints examined was statistically significantly affected: closed arm distance travelled (EPM-CAD) (Figure 5I). EPM-CAD increased by 114.2% in males and decreased by 65.4% in females treated with M8OI compared with sex-matched controls. Statistical comparisons were performed within sex (sex-matched control vs. M8OI). Male–female comparisons within control groups or within M8OI-exposed groups were not assessed statistically; however, inspection of group means indicates marked sex-related baseline differences across EPM measures. For example, in controls, females showed substantially higher closed arm distance travelled than males (EPM-CAD: 8.56 vs. 2.01; ~4.3-fold higher), and markedly higher centre time (EPM-CT: 36.28 vs. 1.02; ~35.7-fold higher), whereas closed arm entries were higher in males (EPM-CAE: 6.83 vs. 1.33; ~5.1-fold higher). Following M8OI exposure, the direction of some sex-related mean differences shifted for selected endpoints, including EPM-CAD (M8OI: males 4.30 vs. females 2.97; ~1.45-fold higher in males) and centre time (M8OI: males 17.18 vs. females 6.92; ~2.5-fold higher in males), consistent with a sex-dependent pattern in the behavioural profile. A formal assessment of sex × treatment interaction was not performed in this study and is beyond the scope of the current analysis.

## 4. Discussion

The mechanism of toxicity of M8OI (or M8OI cations with other innocuous anions such as Br−) has been examined in a range of mammalian cell types, including rat neuroendocrine/neuronal cells [33,36,37]; human skin fibroblasts and colorectal epithelial cells [38] and liver/liver-related cell types [6,7,8,10,11,15,16]. Recent benchmark dose modelling using transcriptomics and metabolomics has also been applied to alkylimidazolium ionic liquid hepatotoxicity, providing quantitative frameworks relevant to health risk assessment [39,40]. The overall conclusions from these studies are that M8OI exposure leads to mitochondrial dysfunction and cell death mechanisms associated with ROS increase and activation of apoptotic pathways. Very high concentrations of M8OI tend to increase cell death by necrotic pathways [17], likely through non-specific membrane effects. There is a range of sensitivities to M8OI observed, and our recent studies have investigated the importance of medium substrates used for energy production by cells as pivotal determinants. Cells routinely cultured in high glucose—primarily those used to model hyperglycaemia (25 mM)—are more resistant to M8OI because they can rely on glycolysis alone for longer through a compensatory survival mechanism. This is exemplified by these studies with SH-SY5Y neuroblastoma cells, which are routinely cultured in high glucose-containing media, and are protected from M8OI toxicity by glucose and are sensitised through replacing glucose with galactose (which forces cells to utilise mitochondrial oxidative phosphorylation for energy production [34]). Although SH-SY5Y cells provide a useful model for mitochondrial bioenergetic assessment, they are a transformed human neuroblastoma cell line and may not fully represent primary human neuronal physiology.

Recent work suggests the key initiating event for M8OI toxicity is an inhibition of complex I through its binding to the ubiquinone binding site [18], which is consistent with the ability of glucose to protect against M8OI toxicity. The limited mechanistic data in this study suggest that the mechanism of cell death in SH-SY5Y cells is likely linked with an inhibition of complex I. It should be emphasised that these in vitro data provide mechanistic context under acute exposure conditions, whereas the in vivo findings reflect chronic exposure and functional behavioural outcomes; therefore, direct equivalence between the two datasets should not be assumed.

Given the brain’s high dependence on glucose as its primary energy substrate, the potential neurobehavioural effects of M8OI in vivo were examined. M8OI (and metabolites) were determined in tissues from a previous study that identified the heart and kidney as target organs for M8OI toxicity. To date, all data indicate that M8OI metabolites are significantly less toxic than the parent M8OI [9]. Given that M8OI is primarily renally cleared [12], the high levels of M8OI in kidney tissues (identified in this study) and pathological changes observed [12] are consistent and contextualise the M8OI levels present in the brain. Even taking into account that different regions of the brain may sequester M8OI, it seems reasonable to conclude that brain levels of M8OI are relatively low and unlikely to give rise to overt cell death in the brain. It should be noted that tissue quantification and behavioural outcomes were obtained from separate cohorts; therefore, the tissue concentrations provide contextual exposure information rather than a direct concentration–behaviour linkage within the same animals.

Behavioural endpoints such as open field and rotarod performance cannot, on their own, prove direct CNS neurotoxicity, as systemic or peripheral effects (e.g., kidney, heart or muscle effects) could contribute to altered performance. Therefore, the observed neurobehavioural changes should be interpreted cautiously in the absence of direct brain pathology or mechanistic endpoints in vivo.

Many drugs and chemicals are kept low in the brain due to the protective effect of the blood–brain barrier, which is associated with active excretion by various transporters expressed on the luminal (apical) membrane of brain capillary endothelial cells [41]. These ATP-dependent efflux transporters export xenobiotics back into the blood, thereby limiting brain penetration and maintaining low steady-state brain concentrations [42]. In addition, tight junctions between endothelial cells restrict paracellular diffusion, so entry into the CNS is largely dependent on passive permeability and/or transporter-mediated uptake, both of which can be counteracted by active efflux [42]. In this respect, it is also known that M8OI is a substrate for transporters (p-glycoprotein and BCRP) in the rat [43], and this likely explains, at least in part, the lower levels of M8OI in the brain.

Despite the apparent low concentrations of M8OI in the brain of orally exposed rats, there was a significant reduction in both exploratory and general activity in both sexes. In contrast, a sex difference was seen concerning head rearing frequency (OFT-RF), where M8OI treatment resulted in a statistically significant reduction in males and a statistically significant increase in females. M8OI treatment, therefore, suppressed motor activity without markedly affecting anxious behaviours. Nevertheless, the in vitro exposures were acute (hours to 24 h), whereas in vivo exposure was chronic (20 weeks), so concentration–effect comparisons should be interpreted cautiously. Accordingly, the SH-SY5Y findings are used here to support mechanistic plausibility (mitochondrial bioenergetic disruption) rather than to infer direct neuronal injury in vivo. It is possible that chronic exposure results in functional effects that do not require overt neuronal loss, such as altered neuronal energetics or synaptic dysfunction, or indirect effects mediated by systemic physiology. The behavioural effects observed at relatively low brain concentrations suggest that there may be additional molecular target(s) for M8OI beyond complex I inhibition in the brain, although complex I activity was not directly measured in vivo in this study. Neurobehavioural effects of M8OI may not be of toxicological concern under normal oral exposure levels, given the high exposure levels required to see these effects. However, should the target be more readily accessible in humans than rats, be more readily accessible in a proportion of the population due to variations in disposition and/or exist elsewhere in the body, then this target could be of more toxicological significance.

## 5. Limitations and Future Directions

A limitation of this study is that mechanistic CNS endpoints were not assessed in vivo (e.g., brain complex I activity, ROS/ATP measures, dopamine levels, neuroinflammation markers, or histological assessment of neuronal integrity). This reflects the study design, in which behavioural testing and archived tissue quantification were conducted in separate cohorts (to minimise additional animal use), and tissue quantification was limited to a subset of animals due to remaining tissue availability; therefore, mechanistic brain endpoints were not collected from the behavioural cohort. The in vivo work utilised a single dose level, and water intake was not recorded; therefore, the mg/kg/day value represents an estimated dose based on drinking water concentration. As perfusion was not performed, residual blood contribution to measured whole-brain concentrations cannot be completely excluded. Finally, the in vitro component used a transformed SH-SY5Y model. Future studies should include multi-dose designs, systematic monitoring of water intake, primary neuronal cultures, region-specific brain quantification, and direct brain mechanistic endpoints (e.g., oxidative stress markers and mitochondrial function) to better define causality.

## 6. Conclusions

Oral exposure of M8OI to rats via drinking water suppressed motor activity without marked anxiogenic behaviours, despite low measured whole-brain concentrations. These in vivo findings are most consistent with functional effects that may not require overt neuronal loss and could involve indirect/systemic contributions. The mechanism(s) of these effects remain unknown and suggest additional pathways for adversity in response to M8OI and other methylimidazolium ionic liquids. The in vitro SH-SY5Y data support mechanistic plausibility for mitochondrial bioenergetic disruption under acute exposure conditions, but should not be taken to directly infer neuronal injury in vivo.

## Figures and Tables

**Table 1:** Concentrations of M8OI and its metabolites in the brain, kidney, and serum of orally exposed male rats. Data are the mean and SD of 3 male rats. Only limited remaining tissue was available for LC–MS/MS screening and quantification; therefore, Table 1 reports *n* = 3 treated animals. Control samples were analysed but M8OI and its metabolites were not detected (*n* = 3; data not included).

	[Metabolite] (nM)			
Organ	**M8OI**	**HO8IM-1**	**HO8IM-2**	**COOH7IM**
Brain	40.23 ± 9.67	6.50 ± 0.15	30.40 ± 11.55	50.19 ± 15.68
Kidney	1205.68 ± 76.32	3051.38 ± 168.00	2928.91 ± 168.94	10,375.14 ± 1340.11
Serum	139.80 ± 32.92	428.77 ± 106.51	320.56 ± 80.62	1583.86 ± 227.15

## Data Availability

The original contributions presented in this study are included in the article/Supplementary Materials. Further inquiries can be directed to the corresponding author.

## Supplementary Materials

The following supporting information can be downloaded at: https://www.mdpi.com/article/10.3390/jox16030113/s1, Figure S1: Representative LC–MS chromatograms for detection of M8OI and metabolites in kidney, serum, and brain samples; Table S1: Individual animal-level concentrations of M8OI and metabolites in rat brain, kidney, and serum following chronic oral exposure.

## Abbreviations

M8OI, 1-octyl-3-methylimidazolium (also referred to as [C8mim][Cl]). Note that the term M8OI is used here to denote the M8OI cation paired with the Cl^−^ anion. This is because a variety of anions can and are used in combination with M8OI. The chloride (Cl^−^) salt was selected because chloride is a common physiological anion present at high concentrations in biological fluids. The addition of M8OI to culture media or dosing solutions at the levels used in these studies is not expected to have any significant physiological or toxicological impact through changes in Cl^−^, thereby ensuring that any effects can be attributed to the M8OI cation. MTT, methylthiazolyldiphenyl-tetrazolium bromide. OCR, oxygen consumption rate. ECAR, extracellular acidification rate. CPZ, chlorpromazine.
