# Supplementary material for: Neurobehavioural Effects of the Methylimidazolium Ionic Liquid M8OI in Rats
Source: J Xenobiot. 2026 Jun 17;16(3):113. doi: 10.3390/jox16030113 (PMC13301412; doi:10.3390/jox16030113)
Supplement: Supplementary file 1 [file jox-16-00113-s001.zip › jox-4266715-supplementary.pdf]

# Neurobehavioural Effects of the Methylimidazolium Ionic Liquid M8OI in Rats

**Tarek M. Abdelghany, Alaa A. Budastour, Ahmed S. Kamel, Sherehan M. Ibrahim, Alex Charlton, Simon Wilkinson, Catherine Arden, Noha F. Abdelkader and Matthew C. Wright**

A)

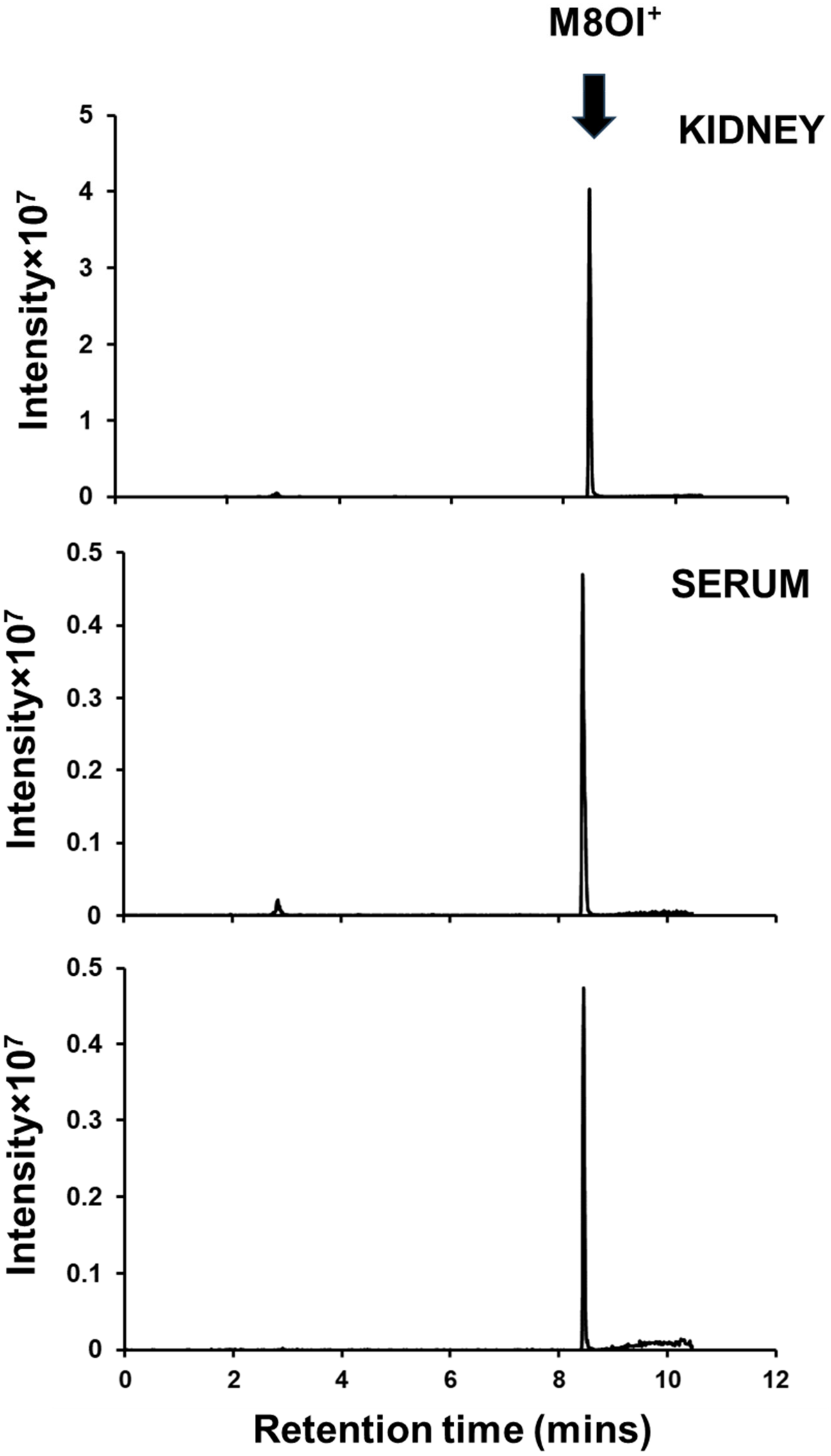

B)

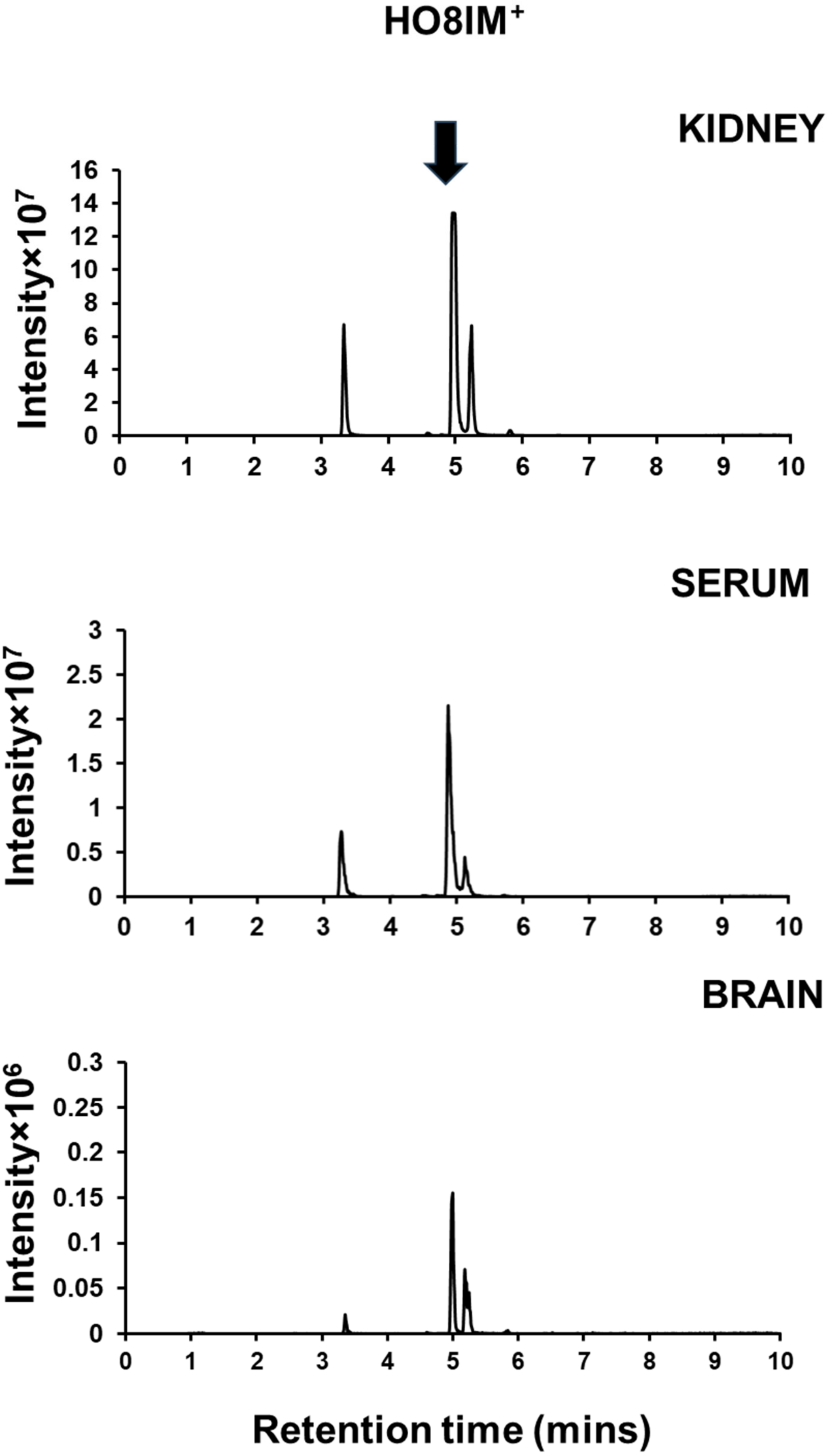
HO8IM+
KIDNEY
Intensity×10^7
16
14
12
10
8
6
4
2
0
0 1 2 3 4 5 6 7 8 9 10
SERUM
Intensity×10^7
3
2.5
2
1.5
1
0.5
0
0 1 2 3 4 5 6 7 8 9 10
BRAIN
Intensity×10^6
0.3
0.25
0.2
0.15
0.1
0.05
0
0 1 2 3 4 5 6 7 8 9 10
Retention time (mins)

C)

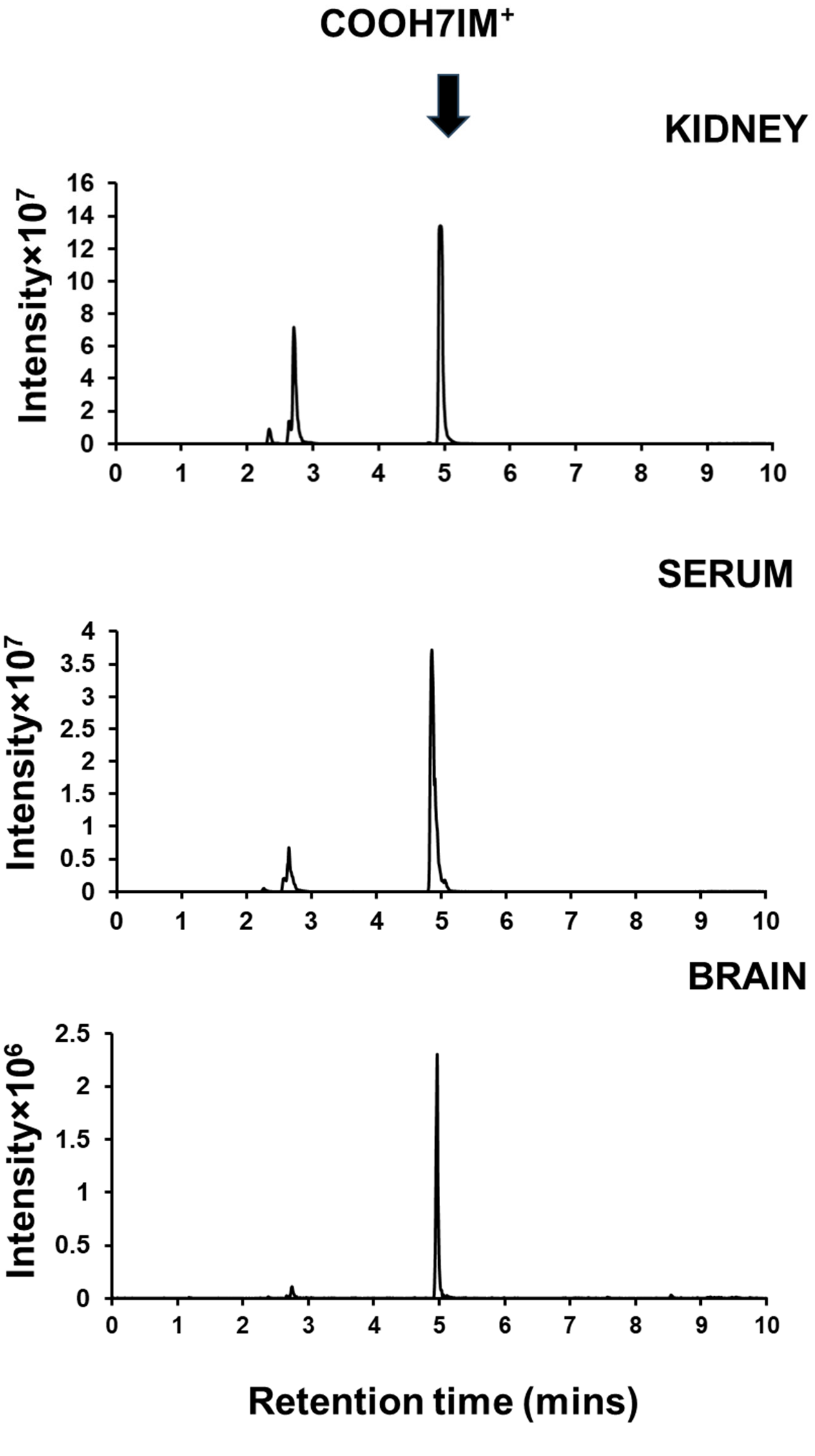
COOH7IM+
KIDNEY
Intensity×10^7
16
14
12
10
8
6
4
2
0
0 1 2 3 4 5 6 7 8 9 10
SERUM
Intensity×10^7
4
3.5
3
2.5
2
1.5
1
0.5
0
0 1 2 3 4 5 6 7 8 9 10
BRAIN
Intensity×10^6
2.5
2
1.5
1
0.5
0
0 1 2 3 4 5 6 7 8 9 10
Retention time (mins)

D)

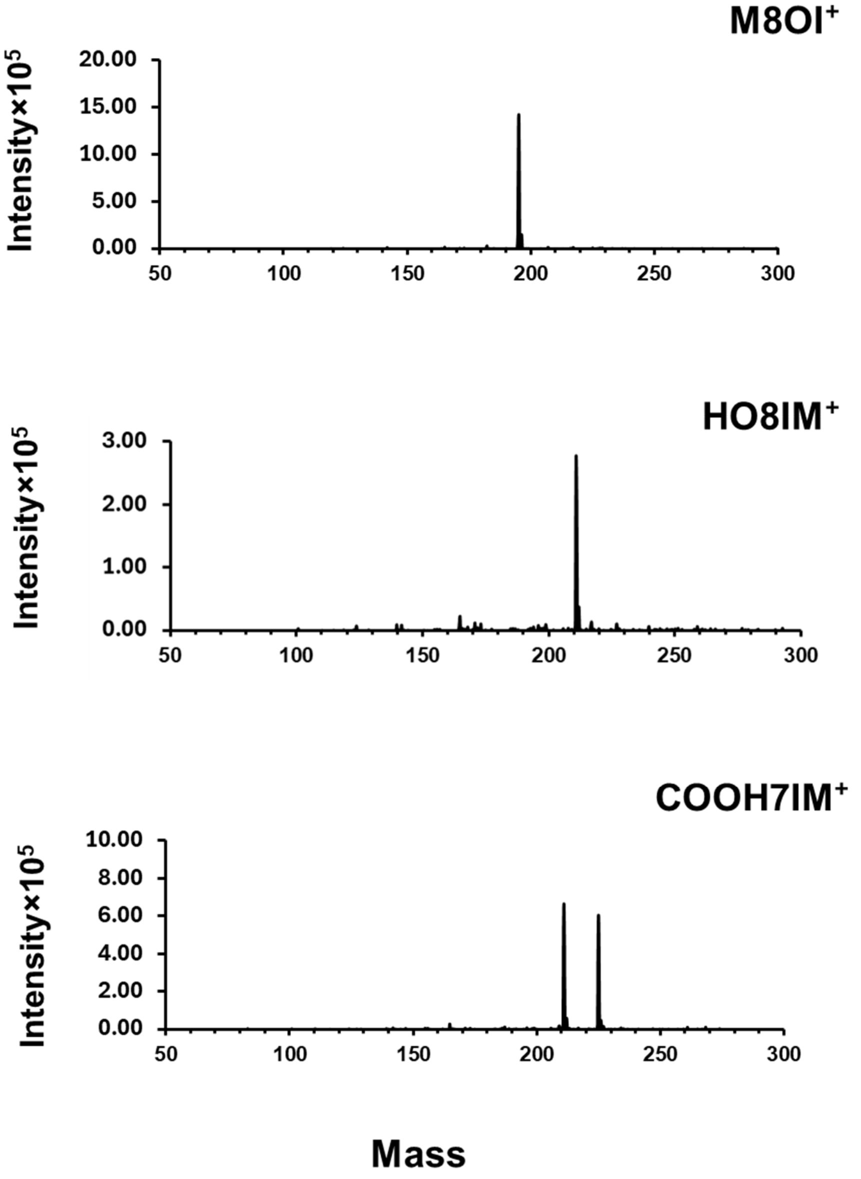


Figure S1. Representative LC–MS chromatograms showing detection of M8OI and metabolites in kidney, serum, and brain samples (MRM intensity versus retention time).A, M8OI$^+$. B, HO8IM$^+$. C, COOH7IM$^+$. D, Intensity versus m/z plots for brain M8OI$^+$ and metabolites.

Table S1. Individual animal-level concentrations of M8OI and metabolites in rat brain, kidney, and serum following chronic oral exposure. Male rats were exposed to M8OI via drinking water (880 mg/L) for 20 weeks (estimated to be equivalent to 44 mg/kg bw/day). M8OI and its metabolites (HO8IM-1, HO8IM-2, and COOH7IM) were quantified by LC–MS/MS in brain, kidney, and serum. Values are reported as individual concentrations (nM) for each animal (Rat 1–3). Abbreviations: M8OI, 1-octyl-3-methylimidazolium; HO8IM, hydroxylated M8OI metabolite; COOH7IM, carboxylated M8OI metabolite.

| Organ | Rat | M8OI (nM) | HO8IM-1 (nM) | HO8IM-2 (nM) | COOH7IM (nM) |
|---|---|---|---|---|---|
| Brain | Rat 1 | 34.22 | 6.66 | 20.35 | 35.98 |
| Brain | Rat 2 | 51.38 | 6.48 | 43.02 | 67.02 |
| Brain | Rat 3 | 35.09 | 6.36 | 27.83 | 47.57 |
| Kidney | Rat 1 | 1293.68 | 3244.83 | 2962.41 | 11277.91 |
| Kidney | Rat 2 | 1165.67 | 2967.13 | 3078.58 | 11012.16 |
| Kidney | Rat 3 | 1157.68 | 2942.18 | 2745.73 | 8835.34 |
| Serum | Rat 1 | 172.81 | 513.30 | 238.90 | 1731.90 |
| Serum | Rat 2 | 106.98 | 309.14 | 322.70 | 1322.33 |
| Serum | Rat 3 | 139.62 | 463.88 | 400.09 | 1697.35 |
